# Primary Pelvic Hydatid Cyst: A Case Report

**DOI:** 10.1155/2011/809387

**Published:** 2011-07-31

**Authors:** Fazl Q. Parray, Shadab Nabi Wani, Sajid Bazaz, Shakeel-ur Rehman Khan, Nighat Shaffi Malik

**Affiliations:** ^1^Department of General Surgery, Sher-i-Kashmir Institute of Medical Sciences, J&K, Srinagar 190011, India; ^2^44-Rawalpora, Govt Housing Colony(sanat nagar), Srinagar 190005, J&K, India; ^3^S.M.H.S. Hospital, Srinagar, India; ^4^Women's College, M. A. Road, Srinagar, India

## Abstract

This is a case report of a young man who presented to us as a case of hypogastric pain and frequency of micturation. General physical examination and radiological evaluation confirmed a multiloculated pelvic swelling. Patient was subjected to laparotomy which confirmed the diagnosis of a primary pelvic hydatid disease. Patient was put on chemotherapy after surgery and is doing well on follow up.

## 1. Introduction

Hydatid disease is a zoonotic parasitic disease most frequently caused by echinococcus granulosus or echinococcus multilocularis. Echinococcus granulosus can reach any organ or tissue of the body where it develops into a small hydatid cyst [1]. The characteristic imaging findings have been described as calcification of the cyst wall, the presence of daughter cysts, or membrane detachment [2]. However the radiologic signs are often nonspecific. Serological tests may be helpful in the diagnosis, but even their reliability is not 100% [3]. Unusual sites of this disease can frequently cause diagnostic problems and lead to diagnostic delays and many potentially serious complications.

Peritoneal hydatidosis could be either primary or more frequently secondary to hydatid cysts in liver or rarely in spleen. Primary peritoneal hydatidosis is rare and has been reported to occur in only 2 percent of all abdominal hydatid disease cases [4]. We report a case of primary hydatid disease of the intraperitoneal pelvic space.

## 2. Case Report

**Figure 1:**
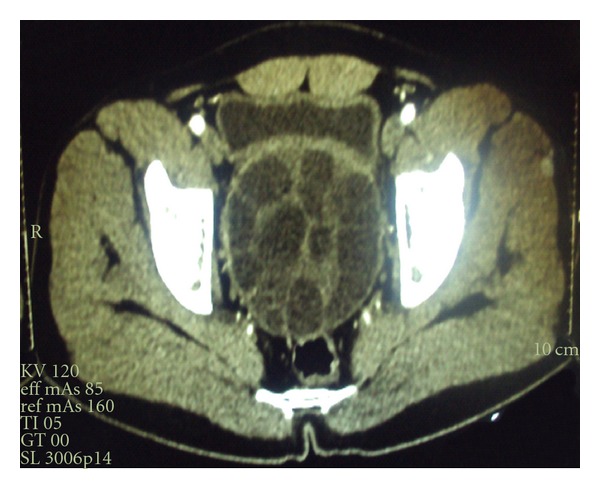
CT showing a pelvic multiloculated hydatid cyst.

**Figure 2:**
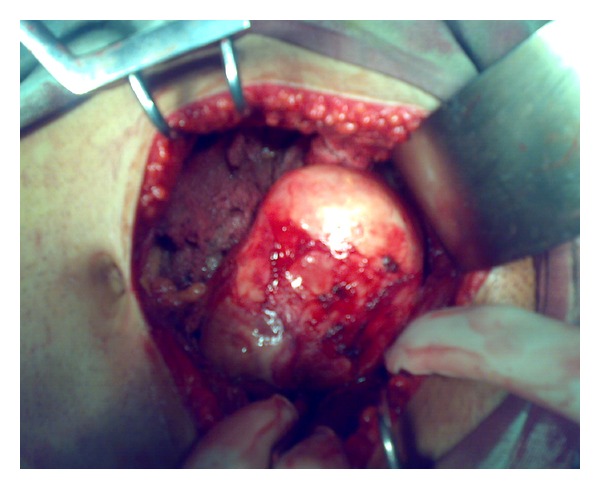
Intraoperative picture of a pelvic hydatid after complete mobilization.

**Figure 3:**
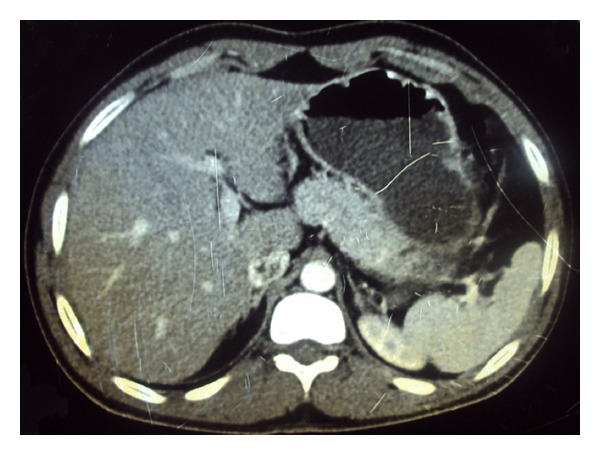
CT showing no involvement of the liver.

A 23-year-old man presented with complaints of dull pain in hypogastric region and frequent micturition for the last 4 months. General physical examination of the patient was unremarkable. Abdominal examination was normal. Digital rectal examination revealed a large, smooth, symmetrical mass lying anterior to the rectum. Ultrasonography of abdomen revealed a large hypoechoic mass with echogenic septations in the pelvis posterior to the urinary bladder. Contrast-enhanced computerized tomogram (CECT) of abdomen and pelvis revealed a huge pelvic cyst 8 × 7 cm in diameter (Figure 1) lying between the urinary bladder and the rectum in the rectovesical pouch. Provisional diagnosis of primary pelvic hydatid disease was made, but hydatid serology was not suggestive of the disease. Radiological examination of chest (chest X-ray PA view) was normal. CECT of the chest is done in our setup only if chest X-ray shows a doubtful lesion in order to decrease the financial burden of the treatment. Exploratory laparotomy revealed a large hydatid cyst in the rectovesical pouch (Figure 2). There were no similar cystic masses in any other abdominal viscera (Figure 3). Cyst was completely excised without any spillage after packing the surrounding area with 1% cetrimide-soaked sponges. Final diagnosis was confirmed by pathological examination. Postoperative period was uneventful. Patient was put on 3 cycles of albendazole therapy; dose of the albendazole was adjusted according to the body weight of the patient. Each cycle of albendazole therapy was of one month duration. After each cycle patient was advised a holiday period of 2 weeks, and in that holiday period liver function and complete blood counts were assessed which in both holiday periods were normal, and subsequently 2nd and 3rd cycles were completed. This is a routine protocol in our department on all patients operated for hydatid cysts. I personally do not advocate laparoscopy in suspected lesions of hydatid disease because of the concern of spillage and it still is not a gold standard for such cysts. The histopathology confirmed it as a hydatid cyst caused by echinococcus granulosus. In spite of the fact that hydatid disease is quite common in our setup, we never encounter a hydatid disease secondary to echinococcus multilocularis.

## 3. Discussion

Hydatid disease or echinococcosis is a parasitic disease caused by infection with larva (metacestode) of the cestode echinococcus. Four species of the genus echinococcus are known to cause infection in humans: echinococcus granulosus (cystic hydatid disease), echinococcus multilocularis (alveolar hydatid disease), echinococcus vogeli, and echinococcus oligarthus (both causing polycystic hydatid disease) [5]. Echinococcus granulosus requires two hosts. Humans become accidental intermediate hosts. The most common site involved is the liver (59–75%), followed in frequency by lung (27%), kidney (3%), bone (1–4%), and brain (1-2%). Other sites such as the heart, spleen, pancreas, omentum, ovaries, parametrium, pelvis, thyroid, orbit, or retroperitoneum, and muscles are very rarely affected [6].

Peritoneal hydatid cyst, either primary or secondary, represents an uncommon but significant manifestation of the disease (approximately 13%). Intraperitoneal hydatid cysts are usually secondary to the rupture (spontaneous or accidental at surgery) of a primary hepatic, splenic, or mesenteric cyst [6]. A solitary cyst in the pelvic cavity can be considered primary only when no other cysts are present. In such a case, the hydatid embryo gains access to the pelvis by hematogenous or lymphatic route. Pelvic hydatid cysts usually present as a nonspecific mass with pressure effects on adjacent organs such as the rectum and urinary bladder. Rarely, they can cause obstructed labour, obstructive uropathy, and renal failure. Sometimes, they can rupture spontaneously [7]. Serology and imaging are the main tools for establishing diagnosis. Ultrasound is the preferred first-line imaging, but CECT gives more precise information regarding the morphology (size, location, neighbourhood, and number) of the cyst. Drug treatment with albendazole has been found to be successful in a proportion of cases, but drug therapy is generally not used as the primary treatment except in cases where the patient is not fit for surgery or the cyst size is smaller or deeply located. Surgery is the most effective treatment. Combination of preoperative albendazole therapy, surgery, and postoperative albendazole therapy is a useful regime. Albendazole suppresses the development of hydatid cysts following intraperitoneal inoculation of protoscolices [7]. En bloc resection without inducing rupture and spreading the daughter cyst is recommended treatment strategy and accepted to be curative for intramuscular hydatid disease [8, 9]. Partial cystectomy, however, is another commonly practiced modality of surgery where the surrounding adhesions or the removal of ectocyst is considered to do more harm than good.

##  Authors' Contribution

F. Q. Parray, first and the corresponding author, worked up and operated upon the patient. S. N. Wani assisted and took all the clinical and radiological photographs. S.-ur-Rehman Khan diagnosed the patient radiologically. N. S. Malik looked for the literature and compiled the study.

##  Conflict of Interests

The authers declare that there is no conflict of interests.

## References

[1] Lewall DB (1998). Hydatid disease: biology, pathology, imaging and classification. *Clinical Radiology*.

[2] Pedrosa I, Saíz A, Arrazola J, Ferreirós J, Pedrosa CS (2000). Hydatid disease: radiologic and pathologic features and complications. *Radiographics*.

[3] Beggs I (1985). The radiology of hydatid disease. *The American Journal of Roentgenology*.

[4] Parray FQ, Gagloo MA, Bhat AH, Chowdri NA, Noor MM (2007). Peritoneal hydatidosis. *The Internet Journal of Surgery*.

[5] Khuroo MS (2002). Hydatid disease: current status and recent advances. *Annals of Saudi Medicine*.

[6] Yuksel M, Demirpolat G, Sever A, Bakaris S, Bulbuloglu E, Elmas N (2007). Hydatid disease involving some rare locations in the body: a pictorial essay. *The Korean Journal of Radiology*.

[7] Seenu V, Misra MC, Tiwari SC, Jain R, Chandrashekhar C (1994). Primary pelvic hydatid cyst presenting with obstructive uropathy and renal failure. *Postgraduate Medical Journal*.

[8] Parray FQ, Ahmad SZ, Sherwani AY, Chowdri NA, Wani KA (2010). Primary paraspinal hydatid cyst: a rare presentation of echinococcosis. *The International Journal of Surgery*.

[9] Arazi M, Erikoglu M, Odev K, Memik R, Ozdemir M (2005). Primary echinococcus infestation of the bone and muscles. *Clinical Orthopaedics and Related Research*.

